# The Vault Complex Is Significantly Involved in Therapeutic Responsiveness of Endocrine Tumors and Linked to Autophagy under Chemotherapeutic Conditions

**DOI:** 10.3390/cancers15061783

**Published:** 2023-03-15

**Authors:** Stefan Bornstein, Igor Shapiro, Alekhya Mazumdar, Kathrin Zitzmann, Svenja Nölting, Edlira Luca, Felix Beuschlein, Ashish Sharma, Constanze Hantel

**Affiliations:** 1 Department of Endocrinology, Diabetology and Clinical Nutrition, University Hospital Zurich (USZ) and University of Zurich (UZH), 8091 Zurich, Switzerland; 2 Medizinische Klinik Und Poliklinik III, University Hospital Carl Gustav Carus Dresden, 01307 Dresden, Germany; 3 Department of Orthopedics, Balgrist University Hospital, 8008 Zurich, Switzerland; 4 Department of Urology, University Hospital Zurich (USZ) and University of Zurich (UZH), 8091 Zurich, Switzerland; 5 Department of Medicine IV, University Hospital, LMU Munich, 80336 Munich, Germany

**Keywords:** autophagy, vault RNA, vault complex, MVP, VPARP, TEP-1, NCI-H295, BON, adrenocortical carcinoma, neuroendocrine tumor, endocrine tumor, EDPM, TNF alpha

## Abstract

**Simple Summary:**

The vault complex, consisting of a major vault protein (MVP), two minor vault proteins (VPARP and TEP1), and small untranslated vault RNA molecules, is considered the largest intracellular ribonucleoprotein particle. Although in recent years vaults were believed to be involved in multidrug resistance (MDR), the exact function of this complex has remained unclear. Our findings reveal a so far unexplored role of the vault complex that is closely linked to the therapeutic responsiveness of endocrine tumors.

**Abstract:**

Cancers display dynamic interactions with their complex microenvironments that influence tumor growth, invasiveness, and immune evasion, thereby also influencing potential resistance to therapeutic treatments. The tumor microenvironment (TME) includes cells of the immune system, the extracellular matrix, blood vessels, and other cell types, such as fibroblasts or adipocytes. Various cell types forming this TME secrete exosomes, and molecules thereby released into the TME have been shown to be important mediators of cellular communication and interplay. Specific stressors in the TME, such as hypoxia, starvation, inflammation, and damage, can furthermore induce autophagy, a fundamental cellular process that degrades and recycles molecules and subcellular components, and recently it has been demonstrated that the small non-coding vault RNA1-1 plays a role as a regulator of autophagy and the coordinated lysosomal expression and regulation (CLEAR) network. Here, we demonstrate for the first time that intra-tumoral damage following effective therapeutic treatment is linked to specific intracellular synthesis and subsequent exosomal release of vault RNAs in endocrine tumors in vitro and in vivo. While we observed a subsequent upregulation of autophagic markers under classical chemotherapeutic conditions, a downregulation of autophagy could be detected under conditions strongly involving inflammatory cascades.

## 1. Introduction

Autophagy is a tightly regulated, catabolic process required for physiological cell homeostasis. It is defined as the fusion of autophagic vacuoles with lysosomes leading to autophagolysosome formation, including subsequent digestion and recycling of cellular components. Activated by specific autophagy-related genes (ATGs), it is induced in response to different stimuli, such as starvation and oxidative stress. In addition, autophagy is of particular importance in cancer where it plays a dual role leading to cytoprotective or cytotoxic effects [1]. Thus, appropriate autophagy modulations could also bare potential for therapeutic strategies against cancer [1,2]. The regulation of autophagy in cancer has been described in response to cytokines, which are related to immune system activation and the immunosuppressive tumor microenvironment (TME); its specific role in this context, however, appears to be highly complex and divergent. Overall, it is known that the regulation of autophagy can influence tumor immunity, suppress the adaptive immune response, and thereby also affect immunotherapies in many ways [3].

An important key player in autophagic flux is the ATG12-ATG5/ATG16 complex, within which ATG5 is indispensable and involved in both canonical and non-canonical autophagy processes. Similarly, LC3BI/II as well as lysosome associated membrane proteins (LAMP1, LAMP2), among others, have been defined as further key factors involved in autophagy. Moreover, long non-coding RNAs and microRNAs have also been shown to modulate autophagy [1,2,4,5,6]. Most recently, the small non-coding vault RNA1-1 has been demonstrated to act as a riboregulator of autophagy and lysosome-mediated chemotherapy resistance [7,8,9,10].

**Figure 1:**
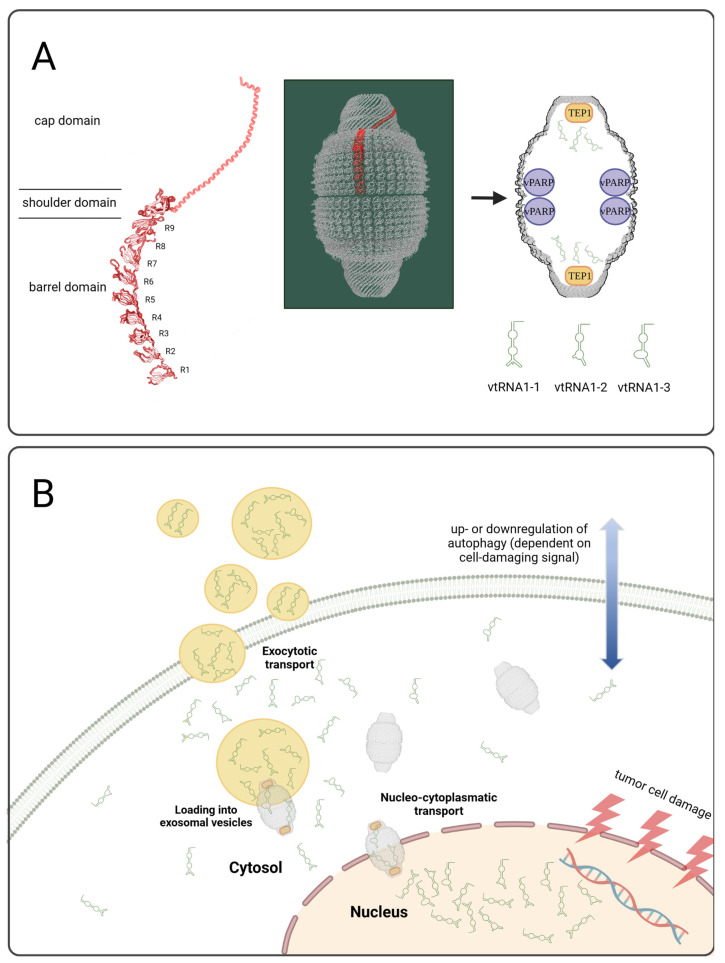
Created with BioRender.com. (**A**) The vault complex and relevant protein and RNA components. MVP structures were adapted from [14,15]. (**B**) Schematic presentation of exosomal vault RNA relsease upon therapeutic tumor cell damage.

Vault particles are the largest ribonucleoprotein complexes identified to date. Bigger than ribosomes and with potential copy numbers of up to 100,000 per cell, vault particles are abundantly expressed and furthermore highly conserved across organisms [10]. The gigantic cellular complex consists of the major vault protein (MVP, accounting for more than 70% of the particle mass and capable of reversible self-assembly [10,11]), vault poly (ADP-ribose) polymerase (VPARP), telomerase-associated protein-1 (TEP1), and the above-mentioned small non-coding vault RNAs (1-1, 1-2, 1-3, and 2-1) from which ≈4.6% are directly associated with the particle while the rest is of cytoplasmic localization [12,13]. However, the exact cellular function of these large and cellular highly abundant vault particles remains largely elusive (Figure 1A).

Here, we demonstrate for the first time that regulation of the whole vault complex, including intracellular upregulation of vault RNAs and their specific exosomal release into the TME, was directly correlated with therapeutic responsiveness in two models of endocrine tumors in vitro and in vivo (Figure 1B). Upon successful tumor tissue damage in all cases, components of the vault complex were increased and vault RNAs were specifically released, accompanying upregulation of certain autophagic markers that could be exclusively observed upon treatment with classical or liposomally encapsulated cytostatics. Conversely, a downregulation of autophagic markers was observed upon treatment with other drug classes directly related to immune system regulation.

## 2. Materials and Methods

### 2.1. Cell Culture, In Vivo Experiments and Gene Array

NCI-H295R and BON cell culture, in vivo experiments, and gene array were performed as previously described in detail [16,17]

### 2.2. Immunofluorescence/Histochemistry

BON and NCI-H295R cells were seeded on 4-well glass chamber slides (Sarstedt, Nümbrecht, Germany) and treated for 2 h with 0.1 µg/mL TNFα on the following day. Cells were fixed with 4% PFA solution (Sigma-Aldrich, St. Louis, MO, USA) and treated with citrate buffer for epitope retrieval. To locate MVP, the mouse anti-MVP antibody (Abcam, Cambridge, UK) and Alexa Fluor488-labeled donkey anti-mouse antibody (Thermo Fisher, Waltham, MA, USA) were used.

Sections (4 µm) of BON and NCI-H295R FFPE tumors from ASA resp. NCI-H295R FFPE tumors in EDPM/LEDPM-treated mice were deparaffinized, rehydrated, and after HIER treatment in citrate buffer, peroxidase was blocked with 0.3% H_2_O_2_. Subsequently, sections were incubated with blocking buffer containing 3% BSA (Roche Diagnostics, Basel, Switzerland), 5% goat serum (Jackson ImmunoResearch Laboratories, West Grove, PA, USA), and 0.5% Tween 20 (Sigma-Aldrich), followed by incubation with rabbit monoclonal anti-LC3B antibody (Abcam) at +4 °C overnight. The secondary antibody used was biotinylated goat anti-rabbit antibody (Vector Laboratories, Newark, CA, USA). Sections were then processed with the Vectastain Elite ABC kit, visualized by DAB, and counterstained with Methyl Green (all three, Vector Laboratories).

### 2.3. EV Isolation by Ultracentrifugation

EVs were purified by differential centrifugation processes, as previously described [18]. Briefly, NCI-H295R and BON cells were seeded into 15 cm dishes in complete medium. After reaching 70–80% confluency, the cells were washed with PBS and subsequently incubated in exosome-depleted FBS for 48 h. The conditioned medium was first centrifuged at 600× *g* for 10 min to remove cells and then centrifuged at 2000× *g* for 15 min to remove debris. The resulting precleared supernatant was then ultracentrifuged in a 70Ti rotor (Beckman-Coulter, Brea, CA, USA) at 10,000× *g* for 30 min at 4 °C and then sequentially ultracentrifuged at 100,000× *g* for 70 min at 4 °C. The supernatant was discarded and the pellet was washed in PBS using the same ultracentrifuge conditions. The pellet containing the purified EVs was collected in PBS buffer and passed through a 0.2 µm pore filter.

### 2.4. EV Isolation by ExoQuick

The ExoQuick kit was used according to the manufacturer’s instructions (System Biosciences, Catalog no. EXOQ5A-1, Palo Alto, CA, USA). In brief, after reaching 70–80% confluency, the NCI-H295R and BON cells were washed with PBS and subsequently incubated in exosome-depleted FBS for 48 h. An initial spin was performed at 10,000× *g* (room temperature) for 10 min to remove cells and debris from each sample, then the corresponding amounts of reagents were added proportional to the starting sample volume, according to the manufacturer’s instructions. Mixtures were vortexed and incubated at 4 °C for up to an hour and then centrifuged at room temperature to precipitate the exosome pellets. Regarding the centrifugation parameters, it was performed for 30 min at 1500× *g*, followed by pellet resuspension in 100 μL PBS buffer. All exosomes were stored at −80 °C immediately after isolation until further NTA analysis.

### 2.5. Nanoparticle Tracking Analysis (NTA)

The size distribution and concentration of nanoparticles in 2D and 3D EV isolations were assessed using the NanoSight NS300 device (Malvern Panalytical, Malvern, UK). Samples were diluted 1:100 in order to obtain an optimal detectable concentration of 40–80 particles/frame.

sCMOS camera levels were kept at 14–15 depending on the concentration of samples. Samples were injected in the 488 nm laser chamber with a constant output controlled by a syringe pump. Five 60 s video recordings were performed for each sample. NTA software (NTA 3.1 Build 3.1.54, Malvern Panalytical, Malvern, UK) was used to analyze the data with a detection threshold of 3. GraphPad Prism (v8.4.2, GraphPad Software, San Diego, CA, USA) was used to integrate the five technical measurements of each sample.

### 2.6. Si-RNA Experiments

NCI-H295R and BON cells (5 million cells/well) were seeded in two 150 cm^2^ flasks per group in penicillin/streptomycin-free medium (day 0). For small interfering RNA (siRNA)-mediated knockdown of MVP, TEP1, and PARP4, cells were transfected with 100 nM of either the targeting or control (siRNA SMARTpool: ON-TARGETplus MVP siRNA #L-004984-01-0020, siRNA SMARTpool: ON-TARGETplus TEP1 siRNA #L-012377-00-0005, siRNA SMARTpool: ON-TARGETplus PARP4 siRNA #L-007244-00-0005, and ON-TARGETplus Non-targeting Pool #D-001810-10-05; Dharmacon, Lafayette, CO, USA) using Lipofectamine RNAiMAX Reagent (#3778030, Invitrogen^TM^, Waltham, MA, USA) in Opti-MEM medium (#31985062 Gibco^TM^, Waltham, MA, USA). On day 2, the cells were induced with 0.1 µg/mL TNFα. On day 3 (48 h after transfection), the cells and culture media were harvested. RNA from the cells was extracted using the RNeasy Mini kit (Qiagen, Hilden, Germany), followed by DNA removal (TURBO DNA-free™ Kit, Thermo Fisher). For cDNA synthesis, 410 ng RNA and the RevertAid™ H Minus First Strand cDNA Synthesis Kit (Thermo Fisher) was used.

### 2.7. RNA Isolation, Reverse Transcription and qRT-PCR

RNA from ASA-treated and control tumors was extracted using the RNeasy Mini kit (Qiagen), followed by DNA removal (TURBO DNA-free™ Kit, Thermo Fisher). For cDNA synthesis, 1.5 µg RNA and the RevertAid™ H Minus First Strand cDNA Synthesis Kit (Thermo Fisher) was used.

For real-time PCR analysis of the ASA-treated BON and NCI-H295R tumors, SsoFast EvaGreen reaction mix (Bio-Rad Laboratories, Hercules, CA, USA) in the MX3000P cycler (Stratagene, La Jolla, CA, USA) was used. The following PCR primers were used: human vault RNA1-1 (forward: 5′-TAGCTCAGCGGTTACTTCGACAGTTCT, reverse: 5′-GGGTCTCGAACAACCCAGACAGG), human vault RNA1-2 (forward: 5′-CGAGTACATTGTAACCACCTCTCTGGG, reverse: 5′-AAGAGCTGGAAAGCACCCGC), and human vault RNA1-3 (forward: 5′-CTCAGCGGTTACTTCGCGTGTCA, reverse: 5′-CGCCCGCGGGTCTCGAACAA). For real-time PCR analysis of the transfected BON and NCI-H295R cells, SsoFast EvaGreen reaction mix (Bio-Rad Laboratories) in the AB7500fast cycler (Applied Biosystems) was used. The following PCR primers were used: human MVP (forward: 5′-GGGTGAGAGTTCCCCATCTG, reverse: 5′-GGCTCACAAGAAGATGACTGGT), human TEP1 (Real Time Primers, Elkins Park, PA, USA; forward: 5′- CCCAAGTCCCTGAACTGTGT, reverse: 5′-ACATTGAAGGCCAAGGTACG), and human PARP4 (Origene, Rockville, DE, USA; forward: 5′-CATGGCGCTTACCTGATGAGTC, reverse: 5′-AACAGTGCCCAGGATGCTGAGT). All gene expression levels in the RNA originated from the cells were normalized to human GAPDH (forward: 5′-AGCCTCCCGCTTCGCTCTCT, reverse: 5′-CCAGGCGCCCAATACGACCA), whereas the exosomal gene expression levels were normalized to miR-15a (forward: Hs_miR-15a_1, Qiagen MS00003178, reverse: miScript Universal Primer, Qiagen).

For real-time PCR analysis of the cell culture supernatants, cells were seeded in 6-well plates and treated with 0.1 µg/mL TNFα on the following day. After 24 h, the supernatants were collected and cleared of cellular debris. Free-floating microRNA was extracted from the supernatant using the Nucleospin RNAII kit (Macherey-Nagel, Düren, Germany). A 12 µL aliquot of the RNA solution and the RevertAid™ H Minus First Strand cDNA Synthesis Kit (Thermo Fisher) and miScript kit (Qiagen) were used for cDNA synthesis. Real-time PCR for vault RNA1-1, vault RNA1-2, and vault RNA1-3 was performed using the SsoFast EvaGreen reaction mix (Bio-Rad Laboratories) in the MX3000P cycler (Stratagene). The miScript miR15a primer (Qiagen) was used for normalization.

### 2.8. Western Blot

#### 2.8.1. BON, NCI ASA Tumors

One million BON and NCI-295R cells per well were seeded in 6-well plates and treated for 2 and 6 h, respectively, with TNFα on the following day. Cells were lysed in the RIPA buffer containing Complete Mini Protease Inhibitor Cocktail (Roche, Basel, Switzerland). The protein concentration was measured using the BCA kit (Thermo Fisher) with the PowerWave340 plate reader (Biotek, Winooski, VT, USA). With 10 µg of proteins loaded per well on the 3.9–20% gel, PAGE was performed for 2 h 30 min at constant voltage of 30 mA per gel in Mini-PROTEAN Tetra Vertical Electrophoresis Cell (Bio-Rad, Hercules, CA, USA).

The eBlot transfer system (Genscript, Piscataway Township, NJ, USA) was used for protein transfer on NC membranes. After blocking with NET-G buffer for 1 h, the membranes were incubated with LC3B antibody (Abcam) at +4 °C overnight, washed, and incubated with the HRP-labeled anti-rabbit antibody (GE Healthcare, Chicago, IL, USA) for 1 h at RT. For visualization, Lumi-Light ECL (Roche) and the LAS3000 imager (Fujifilm, Minato, Tokyo, Japan) were used. Subsequently, membranes were washed, blocked with NET-G, and incubated with mouse β-actin antibody (Sigma-Aldrich) at +4 °C overnight. As the secondary antibody, HRP-labeled anti-mouse antibody (GE Healthcare) was used. β-actin was visualized with Lumi-Light ECL in LAS3000.

#### 2.8.2. NCI EDPM Cells

One million NCI-H295R cells per well were seeded in a 6-well plate and treated with 0.25 x IC50 EDP-M for 24 h starting the following day. Cells were lysed in RIPA buffer containing Complete Mini Protease Inhibitor Cocktail (Roche). The protein concentration was measured using the BCA kit (Thermo Fisher) with the PowerWave340 plate reader. A sample with 20 µg of proteins was loaded on the 3.9–20% gel, which was then run for 4 h 15 min (first at constant voltage of 50 V for 45 min, and then at constant voltage of 200 V for 3 h 30 min) in the Mini-PROTEAN Tetra cell. Protein transfer, incubation with LC3B and β-actin antibodies, visualization, and imaging were performed analogously to the protein samples from ASA-treated tumors. In this setting, for quantification of the LC3B band in Figure 6G the bands were straightened using ImageJ.

#### 2.8.3. BON, NCI TNF Cells

A total of 0.7 m BON and 1 m NCI-295R cells per well were seeded in 6-well plates and treated for 24 h with 0.1 µg/mL TNFα starting the following day. Cells were lysed in RIPA buffer containing Complete Mini Protease Inhibitor Cocktail (Roche). The protein concentration was measured using the BCA kit (Thermo Fisher) with the Tecan Sunrise plate reader (Tecan, Männedorf, Switzerland). A sample with 15 µg of proteins was loaded on Any kD™ Mini-PROTEAN^®^ TGX Stain-Free™ Protein Gels (Bio-Rad, Hercules, CA, USA) and run at constant 100 V until the dye front reached the reference line of the gel. For protein transfer, PVDF membranes and Trans-Blot Semi-Dry Transfer Cell (Bio-Rad) were used. Membranes were cut and after blocking with Blotting-Grade Blocker (Bio-Rad), the upper halves were incubated with mouse anti-MVP antibody (Abcam, Cambridge, UK) and the lower halves were incubated with mouse β-actin antibody (Sigma-Aldrich) at +4 °C overnight, washed, and incubated with HRP-labeled anti-mouse antibody (GE Healthcare). As the ECL substrate, Western Lightning Plus (PerkinElmer, Waltham, MA, USA) was used. The luminescence signal was captured with the Hyperfilm ECL (GE Healthcare).

### 2.9. Statistical Analysis

Statistical analysis and graphical representation of the data were carried out using GraphPad Prism software (version 8, GraphPad Software, La Jolla, CA, USA). If not stated otherwise, comparisons between the control group and two or more treatment groups or between cell lines (mean of each) were performed by one-way ANOVA followed by Bonferroni’s multiple comparisons test. The data are presented in column graphs depicting the mean ± SEM. Statistical significance is denoted as stars in the graphs (* *p* < 0.05; ** *p* < 0.01; *** *p* < 0.001).

## 3. Results

### 3.1. Investigation of Gene Transcripts Related to Therapeutic Responsiveness In Vivo

In a previous study, we investigated the therapeutic potential of the tumor vascular-disrupting agent ASA404 and its downstream mediator TNFα against endocrine tumors in xenograft models for neuroendocrine tumors of the gastroenteropancreatic system (BON) and adrenocortical carcinoma (NCI-H295R). While the BON tumor model demonstrated significant induction of TNFα upon ASA404 treatment and related high therapeutic responsiveness in vivo, no comparable effects were detectable in the NCI-H295R xenografts [16].

**Figure 2:**
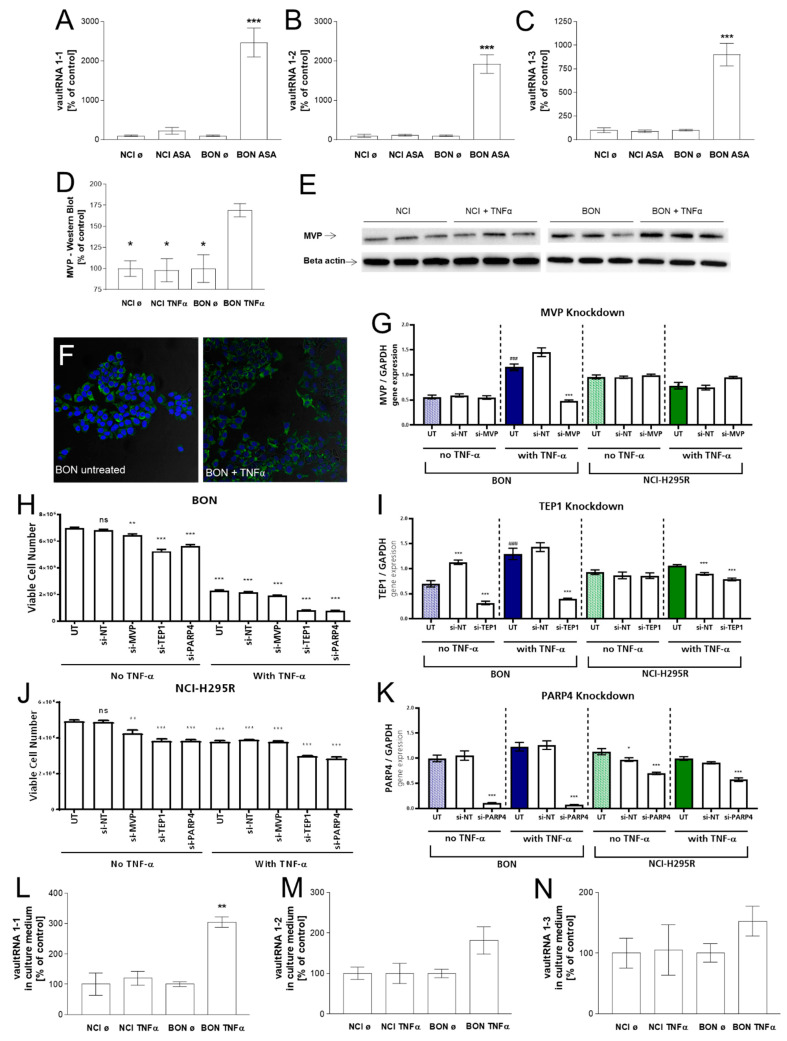
Vault RNA1-1 (**A**), 1-2 (**B**), and 1-3 (**C**) real-time PCR analysis of NaCl- and ASA404-treated BON and NCI-H295R tumors. MVP western blot (**D**,**E**) and MVP immuno-fluorescence (**F**) of TNFα-stimulated BON and NCI-H295R tumor cells. MVP, TEP-1, and PARP4 gene expression analysis (**G**,**I**,**K**) and cell viabilities (**H**,**J**) under TNFα stimulation and specific si-RNA knockdown. Vault RNA levels in cell culture supernatants of TNFα-stimulated BON and NCI-H295R tumor cells (**L**–**N**). The statistical significance is denoted as stars or as rhomb (vs. UT no TNFα) in the graphs (* *p* < 0.05; ** *p* < 0.01; *** *p* < 0.001), ^###^: statistical significance UT no TNF alpha vs. UT with TNF alpha *p* < 0.001. The whole western blots are show in File S1.

The starting point of the current project was the in vivo investigation of specific intra-tumoral gene expression in the therapeutically responding vs. not responding model. Interestingly, a gene-array expression analysis of ASA404-treated BON and NCI tumors revealed that intra-tumoral vault RNA1-1 was almost 17-fold upregulated and thereby the most highly induced transcript upon ASA treatment in the responding BON tumor model (Table 1, [16]). In addition to vault RNA1-1, vault RNA1-2 (5-fold), and vault RNA1-3 (2.5-fold) were found to be significantly upregulated (Table 1). In the next step, we confirmed the specific upregulation of vault RNAs in therapeutically responding BON-tumors in independent real-time PCR analyses, as demonstrated in Figure 2A–C.

### 3.2. Investigation of the Vault Complex in Therapeutically Responding vs. Not-Responding Tumors

To investigate whether the observed regulation was also accompanied by the regulation of other components of the vault complex, we made for further investigations usage of the in this context established therapeutic mediator TNFα as ASA404s initial therapeutic mechanism of vascular disruption cannot be represented in in vitro settings. These experiments revealed that, under these conditions, there was also a significant increase in MVP and TEP-1 but not in PARP4 (VPARP) gene expression upon TNFα treatment in the therapeutically responding model only (Figure 2G–K, blue columns indicate therapeutically responsive BON cells and green columns indicate therapeutically not-responding NCI-H295R cells). Moreover, these effects could be correlated with a significant increase in MVP protein, as demonstrated by immunofluorescence (Figure 2F) and quantified by western blot analysis (Figure 2D,E). To investigate whether a specific reduction in MVP, TEP-1, or PARP4 led to cytotoxic or cytoprotective effects, we also performed si-RNA knockdown experiments, which indicated overall that modulation of these components of the vault complex (Figure 2G–K) might have a general influence on cell viability in both tumor models (Figure 2H,J). Subsequent experiments revealed that TNFα treatment also resulted in a highly significant accumulation of vault RNA1-1 in the cell culture supernatants of BON cells, while no such effect was detectable for therapeutically unresponsive NCI-H295R cells under these conditions (Figure 2L–N).

### 3.3. Investigation of Exosomal Release of TNFα-Treated BON and NCI-H295R Cells

**Figure 3:**
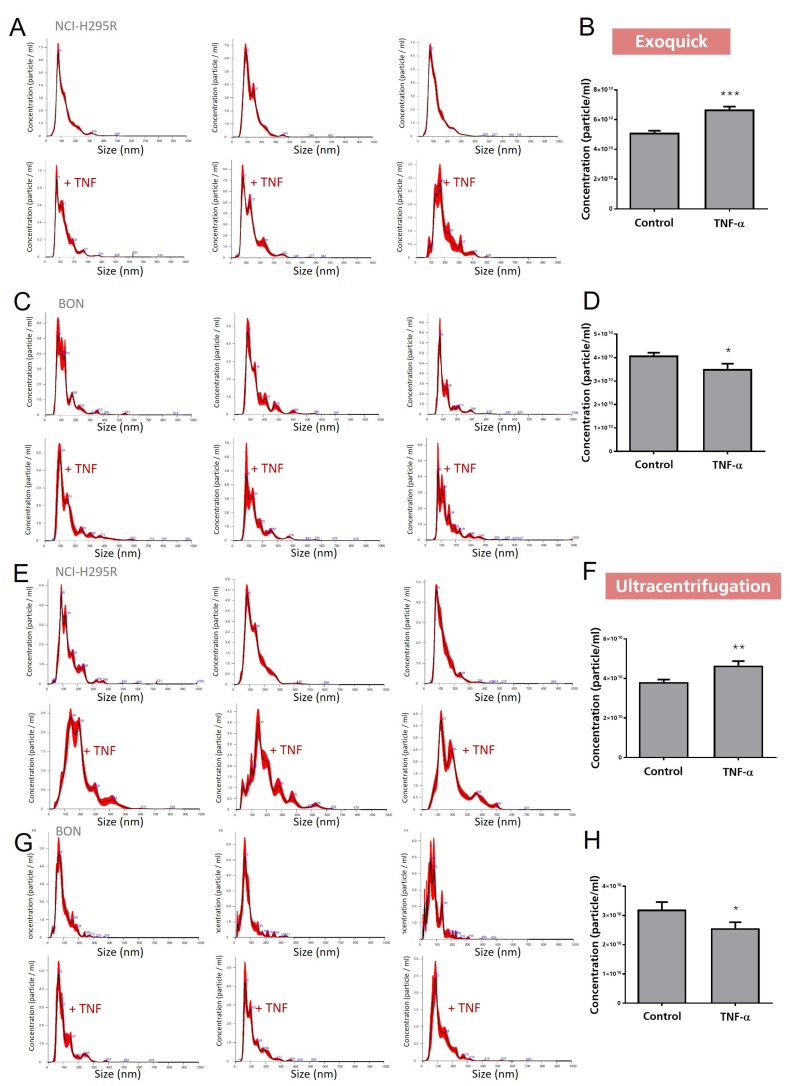
Nanoparticle tracking analysis including size distribution (**A**,**C**,**E**,**G**) and quantification of exosomes isolated by either ExoQuick (**B**,**D**) or ultracentrifugation (**F**,**H**) from cell culture supernatants of TNFα-treated BON and NCI-H295R cells. The statistical significance is based on paired t-tests vs. controls and is denoted as stars (* *p* < 0.05; ** *p* < 0.01; *** *p* < 0.001).

**Figure 4:**
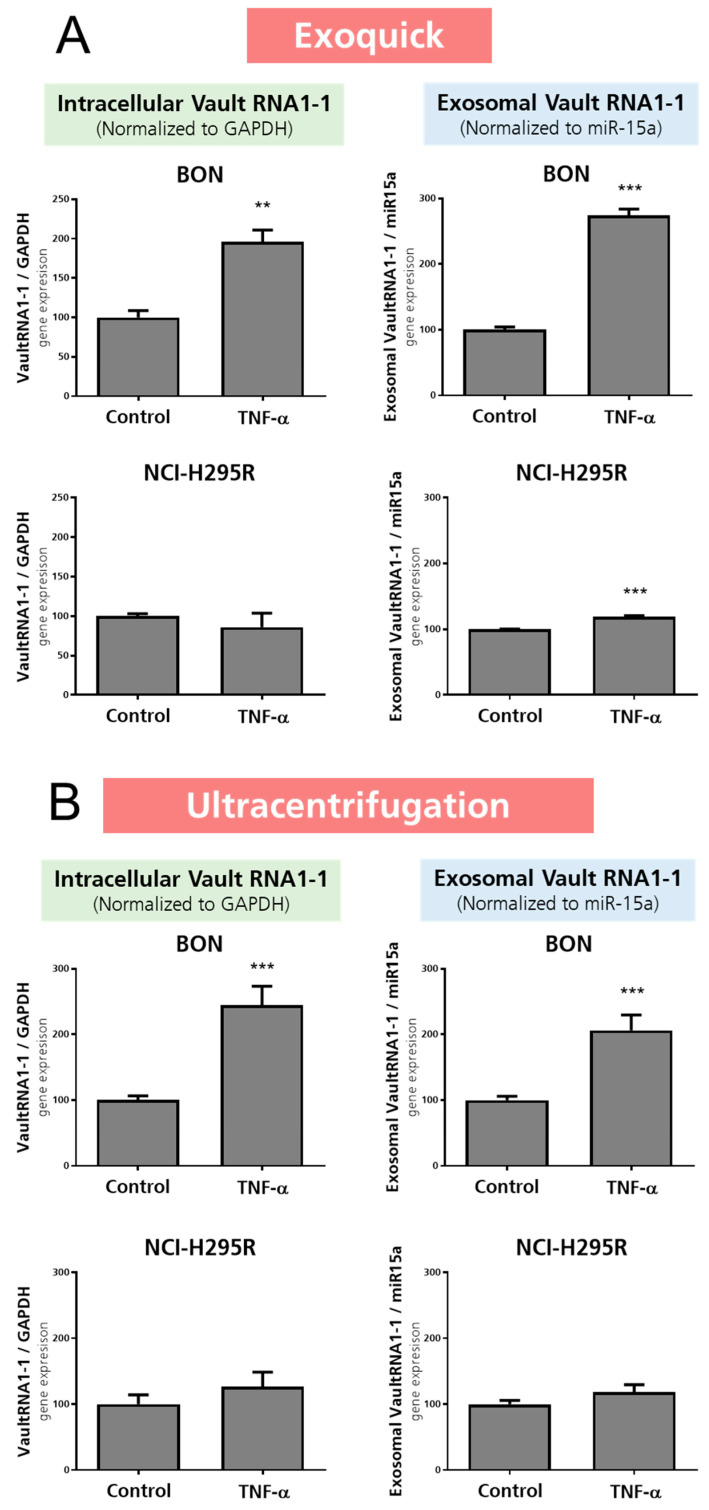
Intracellular and exosomally loaded vault RNA1-1 from cells and cell culture supernatants of TNFα-treated BON and NCI-H295R cells. Exosomes were either isolated by ExoQuick (**A**) or ultracentrifugation (**B**). The statistical significance is based on paired *t*-tests vs. controls and is denoted as stars (** *p* < 0.01; *** *p* < 0.001).

To follow up on the observed specific vault RNA1-1 accumulation in the cell culture supernatants, we next isolated the exosomes from these cell culture supernatants and performed nanoparticle tracking analysis (NTA). NTA allows the investigation of size distribution and relative concentration of extracellular vesicles/mL in cell culture supernatant or bodily fluid. As the methodological gold standard for the isolation of exosomes remains under debate, we further included two standards in our exosome-related experiments in all settings: (a) a kit purification (ExoQuick) and (b) ultracentrifugation. As presented in Figure 3, these experiments revealed successful isolation of extracellular vesicles by both methods, leading to preparations that demonstrated specific peaks in the expected exosomal size range in the subsequent NTA analysis. For the quantification of particle concentration/mL, we included four exemplary pictures representing secreted vesicle quantities. However, it has to be mentioned that no clear correlation between therapeutic responsiveness and quantities of released exosomes was detected overall. In contrast, we found a clear correlation with the loading of vault RNA1-1 content into these exosomes. As depicted in Figure 4, our experiments not only revealed a highly significant increase in intracellularly increased vault RNA1-1 expression, but we also found significantly elevated levels of exosomally released vault RNA1-1 from TNFα-treated BON cells.

### 3.4. Correlation with Autophagic and Lysosomal Markers

**Figure 5:**
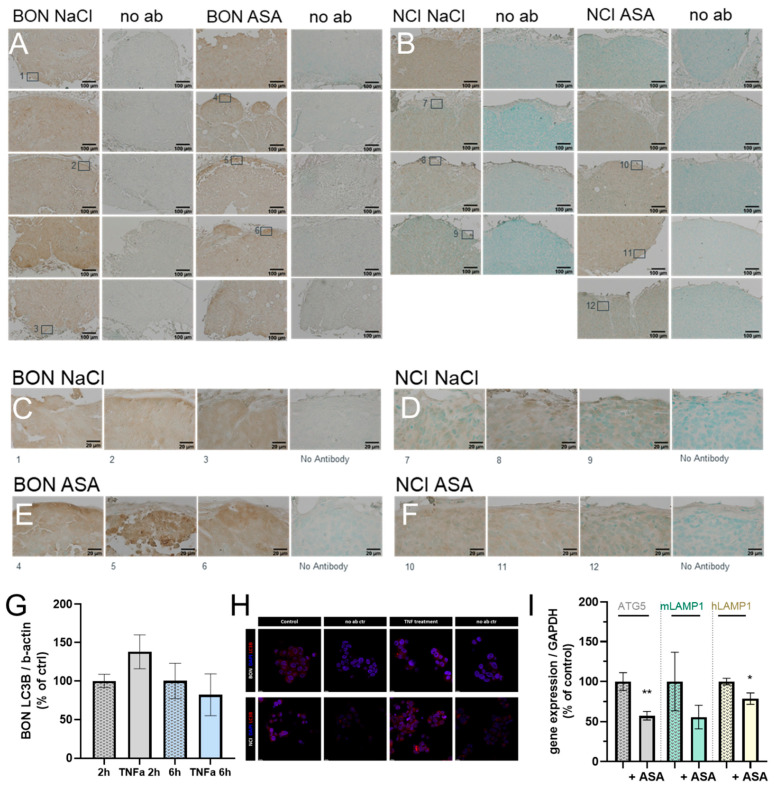
LC3B immunohistochemistry of NaCl- and ASA404-treated BON (**A**,**C**,**E**) and NCI-H295R (**B**,**D**,**F**) tumors. LC3BI Quantification of TNFα-treated BON tumor cells by western blot (**G**) and LC3B visualization by immunofluorescence in vitro (**H**). ATG5, murine LAMP-1, and human LAMP-1 gene expression analysis in NaCl- and ASA404-treated BON tumors in vivo (**I**). The statistical significance is based on paired *t*-tests vs. controls and is denoted as stars (* *p* < 0.05; ** *p* < 0.01).

For correlation with autophagic flux, we first investigated general LC3B abundance and potential occurrence of LC3BII puncta in ASA404-treated BON and NCI-H295R tumors. As depicted in Figure 5, the staining revealed overall for ASA404 treated BON-tumors that some treatment affected tumor regions (Figure 5A), which stained higher for LC3B (Figure 5C) compared to the NaCl-treated controls (Figure 5E), and also in NCI-H295R tumors (Figure 5B,D,F), but did not lead to obviously detectable LC3BII punctae under the chosen analytical setting. To further clarify this point in a more quantifiable setting, we complemented the in vitro setting again using TNF as an important therapeutic mediator of ASA404’s effects. While quantification of LC3B 2 h after TNFα treatment indicated some tendencies (not significant) towards increased protein levels, these tendencies were diminished after 6 h (Figure 5G). Furthermore, no significant changes in autophagic flux were reflected by the detected overall low levels of LC3BII in BON cells. This absence of potential modulation was comparable to that observed in NCI-H295R cells (Supplemental Figure S1). Moreover, the immunofluorescence images confirmed these outcomes microscopically (Figure 5H). Of note, further investigations of human ATG5 and human LAMP1 (hLAMP1) demonstrated a significant downregulation of these markers in ASA-treated BON tumors. Murine LAMP1 (mLAMP1)—representing the lysosomal status of potentially infiltrated host macrophages into the tumor tissue—remained unchanged (Figure 5I).

### 3.5. Proof of Principle in NCI-H295R Tumors and Correlation with Autophagic and Lysosomal Markers upon Chemotherapeutic Treatment

Next, we hypothesized that if treatment-dependent modulation of the vault complex and exosomal release of vault RNAs are general mechanisms of action in affected endocrine tumor tissues, investigations of NCI-H295R tumors under a therapeutic scheme leading to approved tissue damage should reveal further insight.

**Figure 6:**
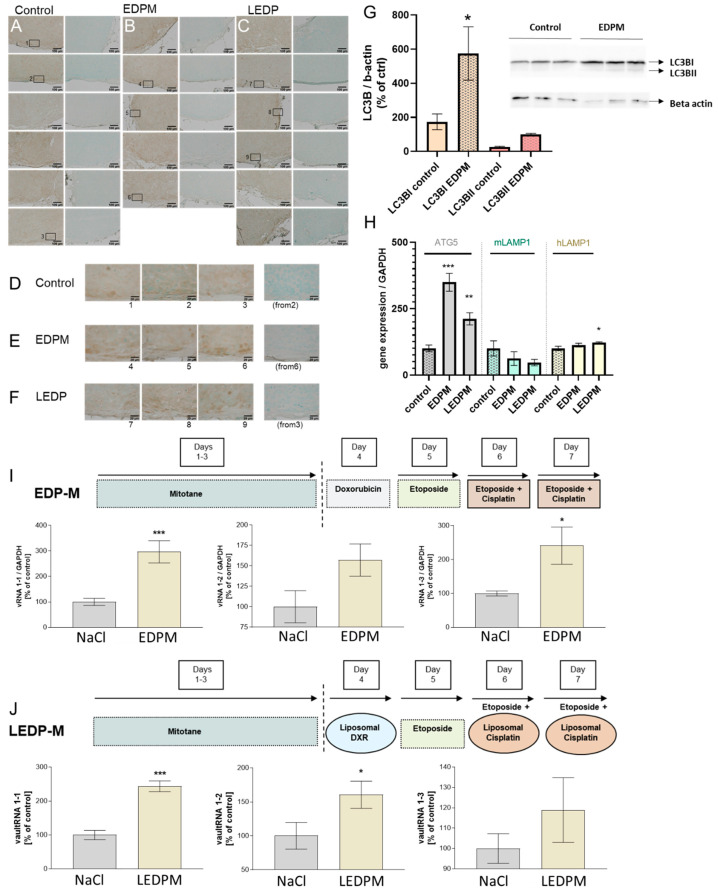
LC3B immunohistochemistry of NaCl- (**A**,**D**), EDPM- (**B**,**E**) and LEDPM- (**C**,**F**) treated NCI-H295R tumors. LC3BI and II quantification of EDPM-treated NCI-H295R tumor cells by western blot in vitro (**G**). ATG5, murine LAMP-1, and human LAMP-1 gene expression analysis in NaCl-, EDPM-, and LEDPM-treated NCI-H295 tumors in vivo (**H**). The statistical significance is based upon one-way ANOVA, including Dunnett-s post-test vs. controls and is denoted as stars (* *p* < 0.05; ** *p* < 0.01; *** *p* < 0.001). Vault RNA1-1, 1-2, and 1-3 real-time PCR analysis of EDPM- (**I**) and LEDPM- (**J**) treated NCI-H295R tumors (two-tailed unpaired *t*-test).

The investigation of EDPM (etoposide, doxorubicin, cisplatin, mitotane)- and LEDPM (etoposide, liposomal doxorubicin, liposomal cisplatin, and mitotane)-treated NCI-H295R tumors had previously demonstrated significant anti-tumoral effects, including histologically proven damage upon both treatment schemes [17,19]. Of note, as demonstrated in Figure 6, real-time PCR analysis under these conditions revealed for the first time a highly significant and treatment-dependent upregulation of vault RNAs also in NCI-H29R tumors (Figure 6I,J). Subsequent immunohistochemical analysis of LC3B again demonstrated some affected areas (Figure 6E,F), which correlated with slightly higher staining intensity compared to control tumors (Figure 6D). However, under the defined microscopic conditions, the obtained images for NCI-H295 tumors did not really cluster in two clearly distinct groups (Figure 6A–C). Thus, for further clarification and optimized quantification, we performed western blotting and real-time PCR analysis again in parallel. In contrast to the previously analysed TNFα-treated tumors, these experiments demonstrated a clear upregulation of autophagic flux. Western blot analysis revealed a significant upregulation of LC3BI in EDPM-treated NCI-H295R cells, which was accompanied by the same tendency for LC3BII (Figure 6G). Following the same line, real-time PCR analysis of EDPM- and LEDPM-treated NCI-H295R tumors showed highly significant upregulation of ATG5 and hLAMP1 (Figure 6H).

## 4. Discussion

Vault particles, with sizes of 13 MDa, are the largest ribonucleoproteins identified to date, and their high copy numbers (10,000–100,000 per cell) and highly conserved compositions suggest fundamental functions in eukaryotic cells [10]. Interestingly, more than 95% of vault RNAs have been shown to be not directly associated with the protein complex [13]. This finding led in past years to the hypothesis that separate or additional functions might exist for vault RNAs. Indeed, most recently, it was demonstrated that vault RNA1-1 binds to the autophagic p62 receptor, which carries a specific LC3-interaction motif and co-localizes with LC3-positive autophagosomes [8,20,21]. Vault RNA1-1 has been shown to inhibit its function in autophagy under conditions of cellular stress and starvation [8,10]. A reduction of vault RNA1-1 levels during starvation is associated with decreased binding to p62 and subsequent promotion of p62 oligomerization and autophagy. So far, it has remained widely unclear to which extent vault RNA regulation and the subsequent modulation of autophagy are involved in further pathological settings such as cancer [8]. However, most recently, it was demonstrated that vault RNA1-1 knockout in human hepatocellular carcinoma cells led to defects in lysosome function, impairment of autophagy-mediated clearance, and subsequently potentiated effects of the multikinase inhibitor sorafenib [7]. Of note, a strong and specific upregulation of vault RNAs, as detected in our experiments upon tumor damage, has been previously described upon different viral infections [22,23]. Interestingly, as a trigger for the specific upregulation under these conditions, the latent membrane protein 1 (LMP1) was identified. LMP1 is a viral relative of the TNF receptor family, which mimics and shares important parts of IL-1 and TNFα-pathways [24,25]. Fittingly, in our study, TNFα was a strong stimulator of vault RNA, MVP, and TEP1 gene expression and also MVP protein expression in the TNFα-responsive BON tumor model. Moreover, upon TNFα treatment, we detected specific exosomal release of vault RNA1-1. Interestingly, it was previously demonstrated that mouse colon cancer cells secrete exosomes containing MVP and that knockout of MVP led to miR-193a accumulation in donor cells instead of exosomes [26]. Our own preliminary data indicated that knockdown of MVP, TEP1, and PARP4 under TNFα-stimulated conditions might also block loading of vault RNA1-1 into exosomes (Supplemental Figure S2). Unfortunately, our initially implemented non-target control in two independent experiments also led to a significant downregulation of vault RNA1-1 loading specifically under TNFα stimulation in the BON tumor model. Therefore, this unspecific effect needs to be further clarified. However, it is well known that the process of recognition and response to non-self nucleic acids, such as siRNA, is governed by the innate immune system leading to induction of interferon and cytokine synthesis [27]. Thus, there is a clear rationale that simultaneous internal and external stimulation of small nucleic acids, including subsequent communications with the innate immune system, might interact here in some way. However, such potentially complex interactions need to be clarified in more detail in further experiments and were out of the scope of the current project.

In the past years, immunotherapy has gained more and more attention, including new immunotherapeutic approaches against cancer. However, side effects are regularly observed, which hampers the successful completion of clinical trials and their approval for clinical use [27]. Immunostimulatory nucleic acids, which are used in immunotherapy, are known to induce the innate immune system, including cytokine and interferon system activation. Moreover, naturally occurring exogenous nucleic acids are also usually involved in pathogen invasion and initiate in a comparable manner to the innate immune response. It is furthermore known that cells signal both via so called pathogen-associated molecular pattern (PAMP) and damage-associated molecular pattern (DAMP) recognition pathways, and as outlined before under both conditions (TNFα- and LMP1-controlled [22]), a significant increase in vault RNAs was detected by us and others. Our experiments further revealed a specific exosomal release of vault RNAs upon tumor cell damage (TNFα-dependent or independent). Thus, it is prudent to speculate that the appropriate release of vault RNA-loaded exosomal vesicles might also have an influence on therapeutic outcomes via interaction with neighboring tumor cells but also with cells of the immune system.

The location of DAMP and PAMP receptors in endosomes and lysosomes, together with the previously described riboregulation of autophagy by vault RNA1-1, indicates roles in clearance and recycling via pathways that modify autophagic flux [8,27,28]. However, while the induction of components of the vault complex was clearly associated with tissue damage in our study, we detected converse regulation of autophagy under different therapeutic settings. While we observed a downregulation of autophagy upon vascular disruption and subsequent local inflammation in BON cells, an upregulation of LC3B, ATG5, and LAMP1 was detected under multi-chemotherapeutic conditions in the NCI-H295R tumor model. Autophagy is known to be divergent and dependent on cancer context, such as tumor stage and therapy, thereby also hindering common clinical applicability of autophagy activators or inhibitors [29]. Our experiments indicated the same conflicting potential for the role of vault RNA1-1 as a riboregulator of autophagy in endocrine cancers, and thus requires further analysis. While a strength of the study was the inclusion of in vitro and in vivo data for two independent tumor entities, a limitation was that only one cell line was implemented for each tumor entity. Further studies including more representative cell lines reflecting broader heterogeneity will be required. Moreover, it is important to note that the scientific focus of this first study was not to show that the observed changes and modulation of the vault complex are the direct intracellular cause for the different therapeutic responsiveness. Instead, we aimed to show that the highly specific occurrence, exosomal release, and potentially also the loading can be rather correlated as kind of result in therapeutically responsive tumors of both types (BON, NCI-H295R). Interestingly, this was dependent on the highly specific therapeutic responsiveness of the tumor types observed upon different therapeutic regimens tested in vitro and in vivo (TNFα, ASA, or EDPM). Accordingly, our study clearly identifies vault RNA1-1 as a highly interesting therapeutic biomarker for endocrine tumors independent of tumor type and therapy but dependent on specific responsiveness, which could be, due to the detected exosomal release of vault RNAs, furthermore isolated from patients’ bodily fluids. Moreover, we aim to investigate the interplay with cells of the immune system in further studies, investigating them as potential recipients of these highly specifically released vault RNA-loaded exosomes. In sum, our findings reveal new insight into a potential implication of the vault complex in the therapeutic responsiveness of endocrine tumors.

## Figures and Tables

**Table 1:** Gene array analysis of NaCl- and ASA404-treated BON and NCI-H295R tumors.

Entrez Gene ID		Abbreviation	RNA Type	BON	NCI
**56664**	**vault RNA1-1**	**VTRNA1-1**	**non-coding**	**16.6**	**1.8**
677823	small nucleolar RNA, H/ACA box 42	SNORA42	non-coding	10.1	
677775	small Cajal body-specific RNA 5	SCARNA5	non-coding	8.2	
677808	small nucleolar RNA, H/ACA box 23	SNORA23	non-coding	8.0	7.6
677774	small Cajal body-specific RNA 1	SCARNA1	non-coding	7.2	3.7
619565	small nucleolar RNA, H/ACA box 52	SNORA52	non-coding	6.6	3.1
677772	small Cajal body-specific RNA 6	SCARNA6	non-coding	6.4	2.6
677806	small nucleolar RNA, H/ACA box 20	SNORA20	non-coding	6.0	3.2
677819	small nucleolar RNA, H/ACA box 37	SNORA37	non-coding	5.7	2.6
677801	small nucleolar RNA, H/ACA box 14A	SNORA14A	non-coding	5.6	1.5
1349	cytochrome c oxidase subunit VIIb	COX7B	coding	5.4	1.8
**56663**	**vault RNA1-2**	**VTRNA1-2**	**non-coding**	**5.0**	**2.3**
26829	RNA, U5E small nuclear 1	RNU5E-1	non-coding	4.9	2.8
26784	small nucleolar RNA, H/ACA box 64	SNORA64	non-coding	4.4	2.8
677797	small nucleolar RNA, H/ACA box 7B	SNORA7B	non-coding	4.4	2.0
619505	small nucleolar RNA, H/ACA box 21	SNORA21	non-coding	4.2	1.9
6044	small nucleolar RNA, H/ACA box 62	SNORA62	non-coding	4.1	
677802	small nucleolar RNA, H/ACA box 14B	SNORA14B	non-coding	4.1	
692148	small Cajal body-specific RNA 10	SCARNA10	non-coding	4.1	2.7
677837	small nucleolar RNA, H/ACA box 60	SNORA60	non-coding	4.1	1.5
574040	small nucleolar RNA, H/ACA box 6	SNORA6	non-coding	4.1	
677811	small nucleolar RNA, H/ACA box 28	SNORA28	non-coding	4.0	2.2
6286	S100 calcium binding protein P	S100P	coding	3.9	
677825	small nucleolar RNA, H/ACA box 44	SNORA44	non-coding	3.9	
692158	small nucleolar RNA, H/ACA box 57	SNORA57	non-coding	3.8	2.0
677781	small Cajal body-specific RNA 16	SCARNA16	non-coding	3.8	
619568	small nucleolar RNA, H/ACA box 4	SNORA4	non-coding	3.6	
26824	RNA, U11 small nuclear	RNU11	non-coding	3.6	
677773	small Cajal body-specific RNA 23	SCARNA23	non-coding	3.5	1.8
85495	ribonuclease P RNA component H1	RPPH1	non-coding	3.4	1.7
26776	small nucleolar RNA, H/ACA box 71B	SNORA71B	non-coding	3.3	
541471	uncharacterized LOC541471	LOC541471	coding	3.3	
100033436	small nucleolar RNA, C/D box 116-25	SNORD116-25	non-coding	3.3	1.8
114599	small nucleolar RNA, C/D box 15B	SNORD15B	non-coding	3.2	1.9
677809	small nucleolar RNA, H/ACA box 24	SNORA24	non-coding	3.2	2.4
401466	chromosome 8 open reading frame 59	C8orf59	coding	3.0	
100151683	RNA, U4atac small nuclear (U12-dependent splicing)	RNU4ATAC	non-coding	3.0	1.6
692225	small nucleolar RNA, C/D box 94	SNORD94	non-coding	3.0	
100033438	small nucleolar RNA, C/D box 116-26	SNORD116-26	non-coding	3.0	2.4
4477	microseminoprotein, beta-	MSMB	coding	2.9	
677829	small nucleolar RNA, H/ACA box 49	SNORA49	non-coding	2.9	1.8
9446	glutathione S-transferase omega 1	GSTO1	coding	2.9	
93081	testis expressed 30	TEX30	coding	2.9	
51642	mitochondrial ribosomal protein L48	MRPL48	coding	2.8	1.9
5203	prefoldin subunit 4	PFDN4	coding	2.8	
677793	small nucleolar RNA, H/ACA box 2A	SNORA2A	non-coding	2.8	
521	ATP synthase, H+ transporting, mitochondrial Fo complex, subunit E	ATP5I	coding	2.8	
3957	lectin, galactoside-binding, soluble, 2	LGALS2	coding	2.8	
4709	NADH dehydrogenase (ubiquinone) 1 beta subcomplex, 3, 12kDa	NDUFB3	coding	2.8	1.6
51503	CWC15 spliceosome-associated protein homolog (S. cerevisiae)	CWC15	coding	2.7	1.7
6750	somatostatin	SST	coding	2.7	
594839	small nucleolar RNA, H/ACA box 33	SNORA33	non-coding	2.7	
25826	small nucleolar RNA, C/D box 82	SNORD82	non-coding	2.7	1.6
6201	ribosomal protein S7	RPS7	coding	2.7	1.9
677770	small Cajal body-specific RNA 22	SCARNA22	non-coding	2.7	2.2
677798	small nucleolar RNA, H/ACA box 9	SNORA9	non-coding	2.7	
26777	small nucleolar RNA, H/ACA box 71A	SNORA71A	non-coding	2.7	1.5
7012	telomerase RNA component	TERC	non-coding	2.7	
677777	small Cajal body-specific RNA 12	SCARNA12	non-coding	2.7	1.6
10247	heat-responsive protein 12	HRSP12	coding	2.6	
100033431	small nucleolar RNA, C/D box 116-20	SNORD116-20	non-coding	2.6	1.6
84300	mitochondrial nucleoid factor 1	MNF1	coding	2.6	1.6
3434	interferon-induced protein with tetratricopeptide repeats 1	IFIT1	coding	2.6	2.8
319103	small nucleolar RNA, C/D box 8	SNORD8	non-coding	2.6	
677814	small nucleolar RNA, H/ACA box 31	SNORA31	non-coding	2.6	
100033821	small nucleolar RNA, C/D box 116-29	SNORD116-29	non-coding	2.6	1.6
4338	molybdenum cofactor synthesis 2	MOCS2	coding	2.6	2.4
29950	SERTA domain containing 1	SERTAD1	coding	2.6	2.1
25906	anaphase promoting complex subunit 15	ANAPC15	coding	2.6	2.0
26828	RNA, U5F small nuclear 1	RNU5F-1	non-coding	2.6	
116937	small nucleolar RNA, C/D box 83A	SNORD83A	non-coding	2.6	
6206	ribosomal protein S12	RPS12	coding	2.6	
51053	geminin, DNA replication inhibitor	GMNN	coding	2.6	
100033420	small nucleolar RNA, C/D box 116-8	SNORD116-8	non-coding	2.6	2.2
57819	LSM2 homolog, U6 small nuclear RNA associated (S. cerevisiae)	LSM2	non-coding	2.6	1.8
677833	small nucleolar RNA, H/ACA box 54	SNORA54	non-coding	2.6	
**56662**	**vault RNA1-3**	**VTRNA1-3**	**non-coding**	**2.5**	
119392	SWI5-dependent recombination repair 1	SFR1	coding	2.5	1.6
341	apolipoprotein C-I	APOC1	coding	2.5	
30836	deoxynucleotidyltransferase, terminal, interacting protein 2	DNTTIP2	coding	2.5	1.8
8365	histone cluster 1, H4h	HIST1H4H	coding	2.5	
6643	sorting nexin 2	SNX2	coding	2.5	2.7
200916	ribosomal protein L22-like 1	RPL22L1	coding	2.5	
26804	small nucleolar RNA, C/D box 45B	SNORD45B	non-coding	2.5	−2.7
1347	cytochrome c oxidase subunit VIIa polypeptide 2 (liver)	COX7A2	coding	2.5	1.5
174	alpha-fetoprotein	AFP	coding	2.5	

## Data Availability

All data that were needed to evaluate the conclusions in the paper are present in the paper.

## Supplementary Materials

The following supporting information can be downloaded at: https://www.mdpi.com/article/10.3390/cancers15061783/s1, Figure S1: MVP and Beta-actin Western Blots of control and TNF alpha treated BON and NCI-H295R cells. LC3B und Beta-actin Western Blots of BON and NCI-H295R control (BC, NC) or TNF alpha (BT, NT) treated cells. LC3B und Beta-actin Western Blots of control and EDPM treated NCI-H295R cells; Figure S2: Differential loading of vault RNA 1 under various si-RNA knockdown conditions for control and TNF alpha treated BON and NCI-H295R cells.
