# Supplementary material for: The Vault Complex Is Significantly Involved in Therapeutic Responsiveness of Endocrine Tumors and Linked to Autophagy under Chemotherapeutic Conditions
Source: Cancers (Basel). 2023 Mar 15;15(6):1783. doi: 10.3390/cancers15061783 (PMC10046419; doi:10.3390/cancers15061783)
Supplement: Supplementary file 1 [file cancers-15-01783-s001.zip › cancers-2263779-supplementary.pdf]

Supplementary Figure S1: MVP and Beta-actin Western Blots of control and TNF alpha treated BON and NCI-H295R cells. LC3B und Beta-actin Western Blots of BON and NCI-H295R control (BC, NC) or TNF alpha (BT, NT) treated cells. LC3B und Beta-actin Western Blots of control and EDPM treated NCI-H295R cells.

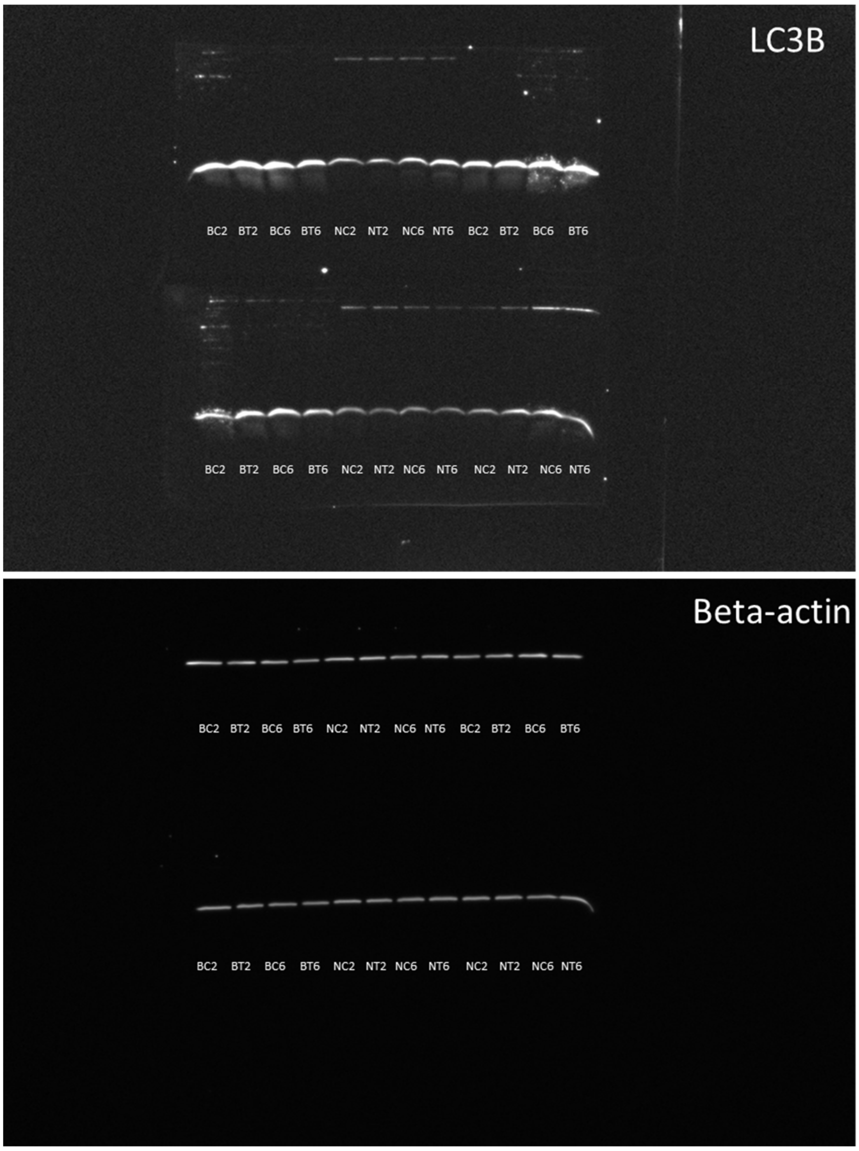

Supplementary Figure S2: Differential loading of vault RNA 1 under various si-RNA knockdown conditions for control and TNF alpha treated BON and NCI-H295R cells.

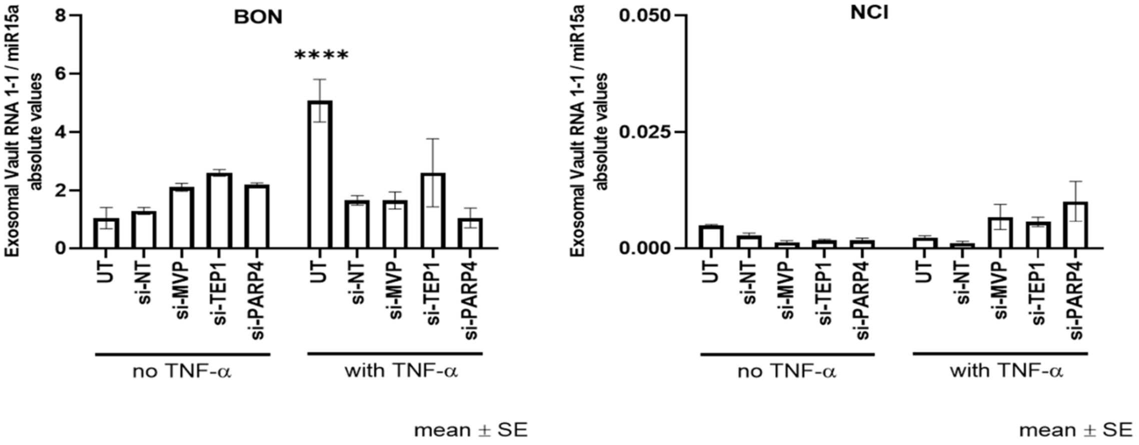

File S1: The whole western blots
The Original WB of Figure 1

LC3B NCI,BON+TNFa

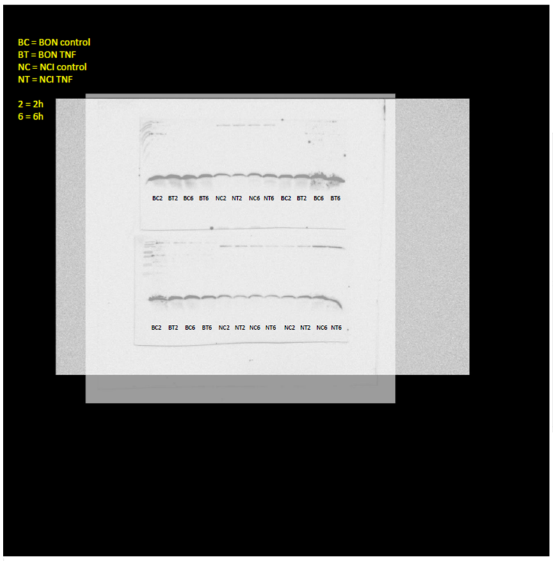

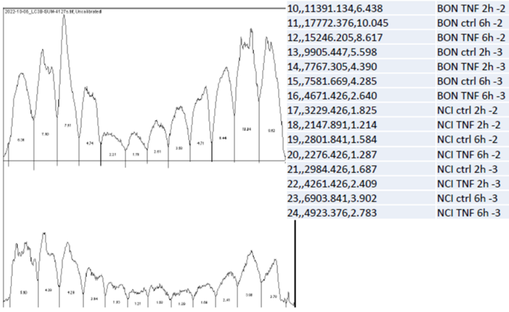

,Label,Area,Percent	
1,,11248.083,6.357	BON ctrl 2h -1
2,,13802.255,7.801	BON TNF 2h -1
3,,13294.426,7.514	BON ctrl 6h -1
4,,8380.719,4.737	BON TNF 6h -1
5,,3911.962,2.211	NCI ctrl 2h -1
6,,3148.012,1.779	NCI TNF 2h -1
7,,4611.719,2.607	NCI ctrl 6h -1
8,,6337.719,3.582	NCI TNF 6h -1
9,,8332.134,4.709	BON ctrl 2h -2
10,,11391.134,6.438	BON TNF 2h -2
11,,17772.376,10.045	BON ctrl 6h -2
12,,15246.205,8.617	BON TNF 6h -2
13,,9905.447,5.598	BON ctrl 2h -3
14,,7767.305,4.390	BON TNF 2h -3
15,,7581.669,4.285	BON ctrl 6h -3
16,,4671.426,2.640	BON TNF 6h -3
17,,3229.426,1.825	NCI ctrl 2h -2
18,,2147.891,1.214	NCI TNF 2h -2
19,,2801.841,1.584	NCI ctrl 6h -2
20,,2276.426,1.287	NCI TNF 6h -2
21,,2984.426,1.687	NCI ctrl 2h -3
22,,4261.426,2.409	NCI TNF 2h -3
23,,6903.841,3.902	NCI ctrl 6h -3
24,,4923.376,2.783	NCI TNF 6h -3

b-actin NCI,BON+TNFa

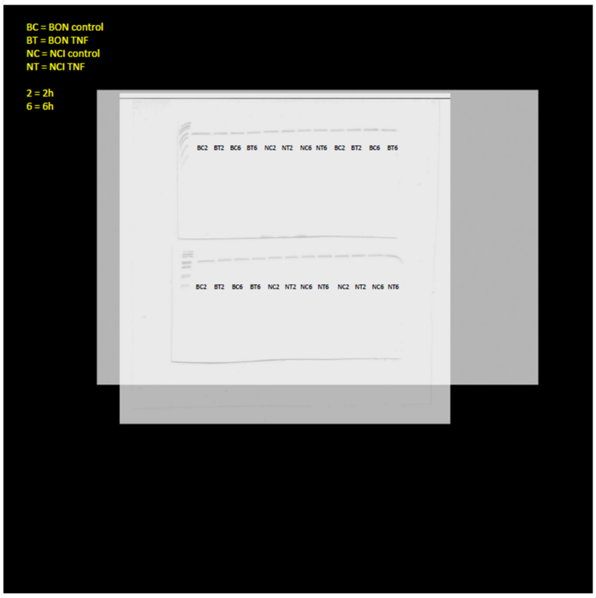

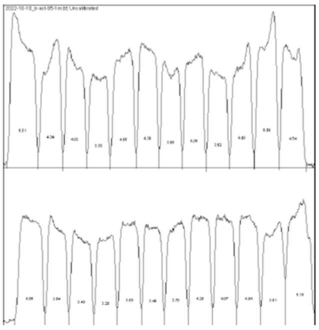

Label,Area,Percent	
1,20212.033,6.505	BON ctrl 2h -1
2,13497.841,4.344	BON TNF 2h -1
3,12489.012,4.020	BON ctrl 6h -1
4,10357.012,3.333	BON TNF 6h -1
5,14145.548,4.553	NCI ctrl 2h -1
6,13648.598,4.393	NCI TNF 2h -1
7,11437.598,3.681	NCI ctrl 6h -1
8,12708.012,4.090	NCI TNF 6h -1
9,10943.305,3.522	BON ctrl 2h -2
10,12662.134,4.075	BON TNF 2h -2
11,16114.134,5.186	BON ctrl 6h -2
12,14713.669,4.736	BON TNF 6h -2
13,15086.497,4.856	BON ctrl 2h -3
14,11319.719,3.643	BON TNF 2h -3
15,10667.841,3.433	BON ctrl 6h -3
16,9949.891,3.202	BON TNF 6h -3
17,11904.719,3.832	NCI ctrl 2h -2
18,10847.891,3.491	NCI TNF 2h -2
19,11504.719,3.703	NCI ctrl 6h -2
20,13054.719,4.202	NCI TNF 6h -2
21,12658.719,4.074	NCI ctrl 2h -3
22,12557.426,4.042	NCI TNF 2h -3
23,12141.426,3.908	NCI ctrl 6h -3
24,16079.205,5.175	NCI TNF 6h -3

The Original WB of Figure 5 G

LC3B NCI EDPM

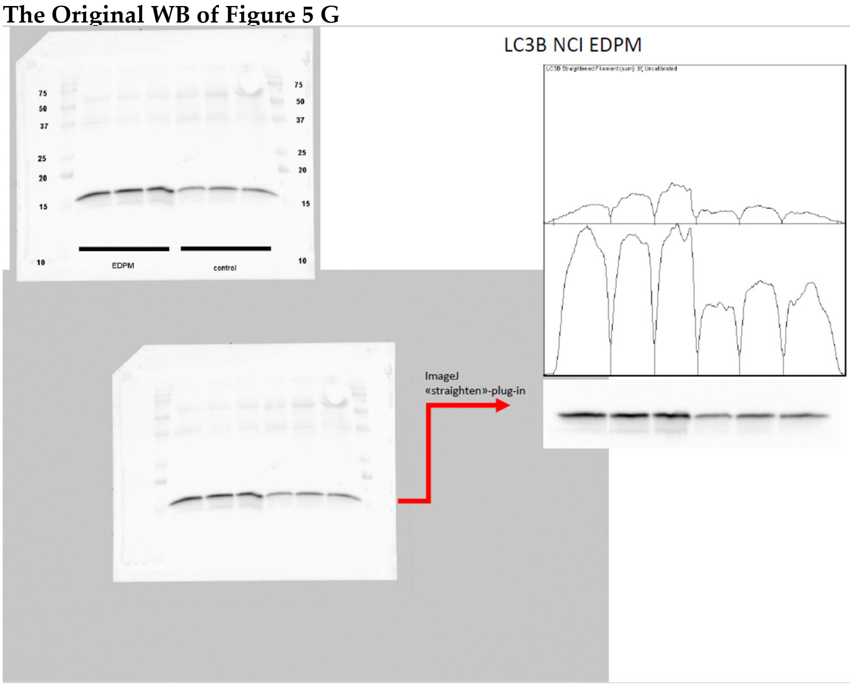

Area	
3549.569	EDPM1 low
4907.79	EDPM2 low
5725.74	EDPM3 low
2240.719	ctrl1 low
2746.598	ctrl2 low
2067.719	ctrl3 low
28155.388	EDPM1 up
23586.104	EDPM2 up
24038.347	EDPM3 up
11938.983	ctrl1 up
15629.933	ctrl2 up
16969.146	ctrl3 up

b-actin NCI EDPM

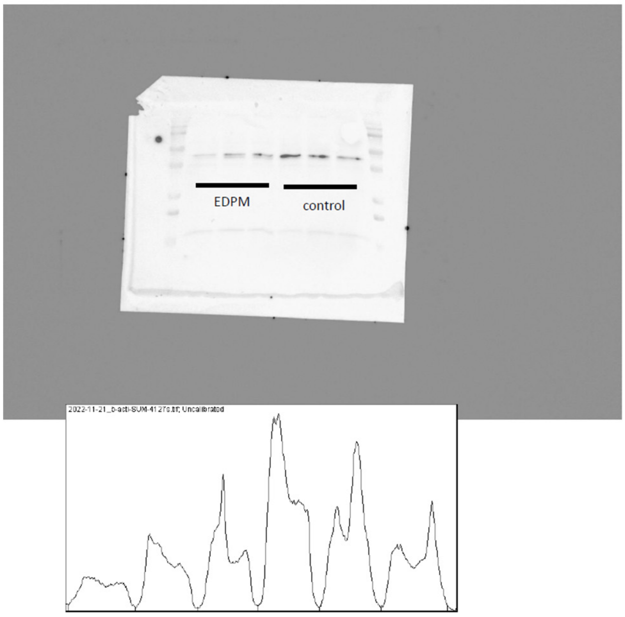

,Area	
1,4284.640	EDPM1
2,6975.882	EDPM2
3,8408.589	EDPM3
4,16801.418	ctrl1
5,12402.832	ctrl2
6,8927.660	ctrl3

The Original WB of Figure 1 E

MVP NCI,BON +TNFa

MVP

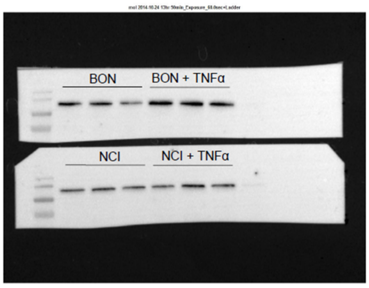

b-actin

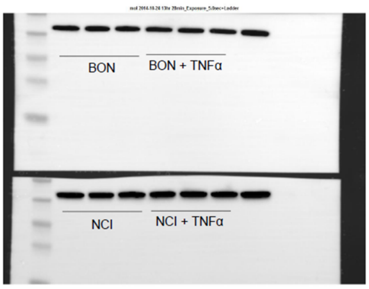

	MVP	B actin		
Gel 1	9'608'317	16'465'903		BON -
	9'196'146	16'492'660		BON -
	5'170'246	15'679'075		BON -
	14'517'409	16'184'317		BON +
	13'546'288	16'387'903		BON +
	12'040'459	15'743'782		BON +
	291'435	20'054'853		MCF-7
Gel 2	7'916'075	19'865'924		NCI -
	9'431'560	19'299'217		NCI -
	7'962'024	22'282'945		NCI -
	7'214'782	22'922'602		NCI +
	12'180'752	23'848'430		NCI +
	9'134'267	23'126'137		NCI +
	315'192	26'844'480		MCF-7
